# Key role of adsorption site abundance in the direct electrochemical co-detection of estradiol and dopamine

**DOI:** 10.1186/s11671-024-04092-8

**Published:** 2024-08-28

**Authors:** Naela Delmo, Ishan Pande, Emilia Peltola

**Affiliations:** 1 Department of Mechanical and Materials Engineering, Faculty of Technology, University of Turku, 20500 Turku, Finland; 2 Department of Electrical Engineering and Automation, School of Electrical Engineering, Aalto University, 00076 Aalto, Finland

## Abstract

**Abstract:**

Estradiol (E2) is a hormone that influences various aspects of women’s health. Beyond its reproductive functions, E2 impacts neurotransmitter systems such as dopamine (DA). Vertically aligned carbon nanofibers (VACNFs) have shown good sensitivity, selectivity against ascorbic acid (AA) and uric acid (UA), biocompatibility, and reduced fouling in DA sensing. In this study, we explore the use of Ti-Ni-CNF electrodes with CNFs grown for 5 min and 30 min for the direct electrochemical co-detection of E2 and DA. The longer growth time led to a 142% increase in average CNF length and a 36% larger electroactive surface area. In E2 detection, the electrodes demonstrate a wide linear range of 0.05–10 µM and sensitivity of 0.016 and 0.020 µA/µM for Ti-Ni-CNF-5 min and Ti-Ni-CNF-30 min, respectively. The sensor performance remains largely unaffected even in the presence of other steroid hormones such as progesterone and testosterone. Co-detection of equimolar E2 and DA shows promising peak separation of 0.34 ± 0.01 V and repeatability after 10 measurements. A notable improvement in the E2/DA peak current ratio, from 0.53 ± 0.07 to 0.81 ± 0.16, was achieved with the increased CNF length. Our results demonstrate the influence of adsorption sites in electrochemical detection, especially for analytes such as E2 and DA that both rely on adsorption for oxidation. While detecting small and fluctuating physiological concentrations remains a challenge, these findings can be used in choosing and fabricating electrode materials for more accurate and accessible continuous hormone measurements, including the possibility of multianalyte sensing platforms.

**Graphic abstract:**

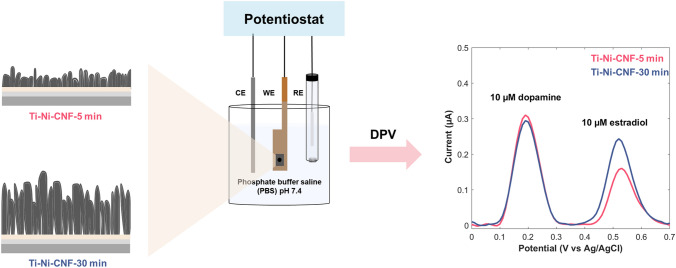

**Supplementary Information:**

The online version contains supplementary material available at 10.1186/s11671-024-04092-8.

## Introduction

Research emphasizes the critical role of considering sex as a variable in health research, particularly for women often underrepresented and historically excluded [1–4]. While a vast amount of work has been done on sensing biomolecules in the body, such as glucose [5], cortisol [6], and dopamine (DA) [7], accurately measuring sex hormones remains a challenge. Estrogen (E), progesterone (P4), luteinizing hormone, and follicle-stimulating hormone have extremely low and fluctuating concentrations throughout the menstrual cycle [8].

**Fig. 1:**
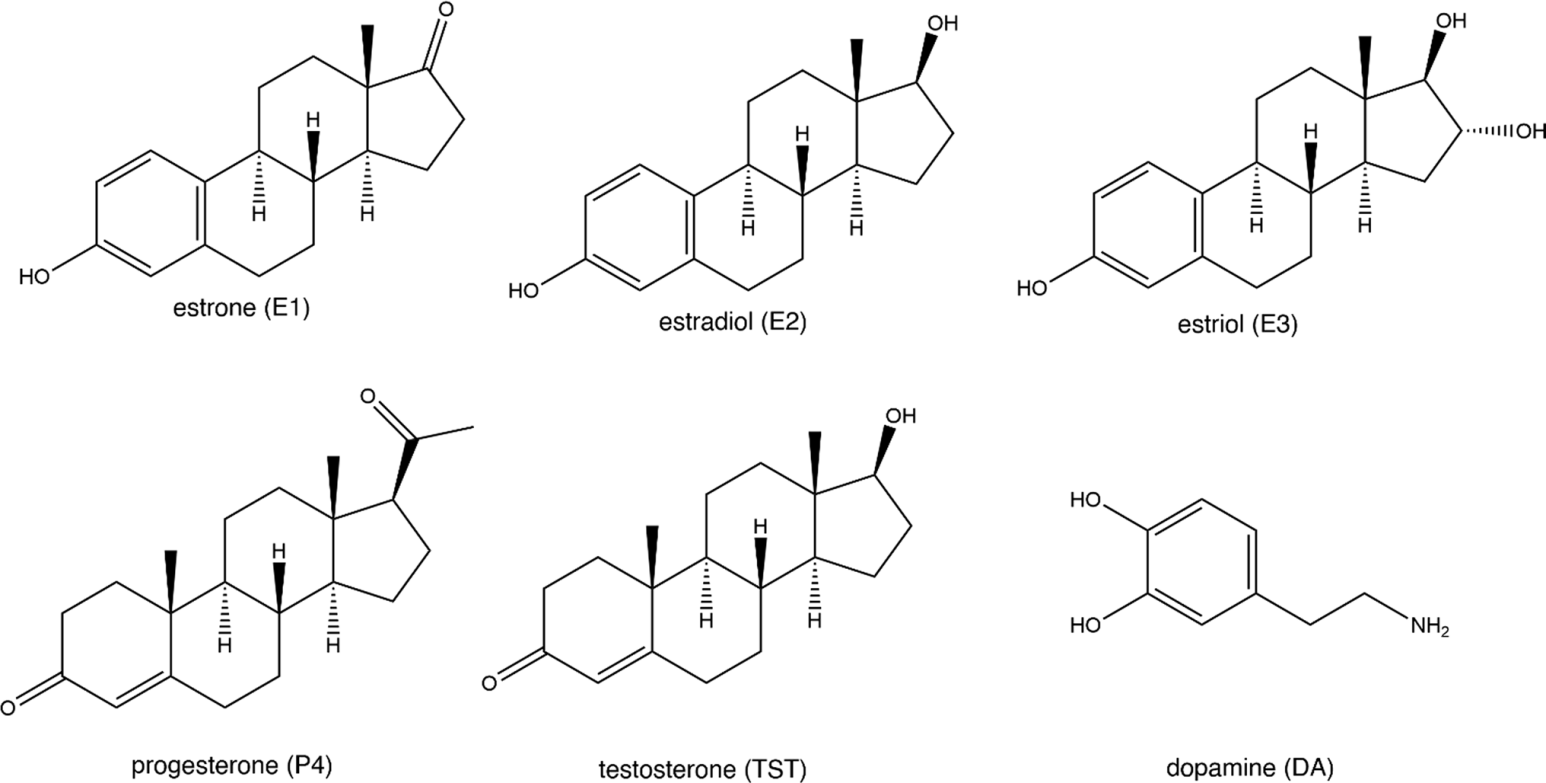
Chemical structures of some steroid hormones and the neurotransmitter dopamine

Estrogen exists in three major forms: estrone (E1), estradiol (E2) and estriol (E3), numbered according to the amount of hydroxyl groups in its structure (Fig. 1). 17-β-estradiol (E2) is the most potent and abundant in premenopausal women [9] functioning not only as a reproductive hormone but also involved in other physiological pathways, including the nervous system [10–13]. Primarily produced in the gonads, the lipophilic E2 can easily move around the body and even cross the blood–brain barrier [14]. Fascinatingly, E2 can also be produced in the brain via de novo synthesis from cholesterol [13, 15]. It exhibits neuroregulatory effects on dopamine (DA) [16, 17] and has been proven to influence the onset of some neurological diseases [18]. Unfortunately, our information about the properties and roles of brain-derived estradiol is limited, with an exceedingly small percentage of neuroscience studies on women [19], definitively highlighting the need for further studies.

Conventional techniques for hormone measurements include immunoassays [20–22] and liquid chromatography (LC) coupled with mass spectrometry (MS) [23, 24]. LC–MS/MS has been used to establish E2 reference intervals for premenopausal and postmenopausal women, revealing E2 blood serum levels of 0.031–2.864 nM and < 0.026 nM, respectively [24]. The same technique was used to determine E2 levels in female rats, from 0.008 to 0.120 nM in blood plasma, and a more stable and higher range of 2 nM in the hippocampus [25]. With numerous factors affecting hormone profiles such as age, sex, and lifestyle, data on these reference E2 concentrations remain limited [24], especially considering the cost, accessibility, and expertise needed.

Electrochemical sensors have emerged as promising healthcare devices with many attractive properties such as simplicity, fast response, ease of miniaturization, and low cost [26, 27]. This offers a way to gather more data and establish more accurate hormone reference values among different population groups. However, the complexity of biological matrices and the presence of multiple interfering compounds are consistent issues for measurements in vivo. Moreover, the polymerization of electroactive compounds like E2, P4, and DA can cause electrochemical fouling [28–30], while the formation of protein and/or lipid leads to biofouling, both of which affect electron transfer during the electrochemical detection.

Carbon nanomaterials, renowned for its high electrical conductivity, high surface area, wide potential window, and cost-effectiveness, have been extensively used in hormone sensing [31–34]. In the absence of biological recognition elements, some E2 sensors have exhibited limits of detection (LODs) in the lower nanomolar levels [35–37], wide linear range [38], and high sensitivity [39]. Furthermore, studies have shown that more complex nanostructures are less susceptible to surface fouling [40, 41]. However, with the rapid E2 signalling on neurons [42] like DA, the temporal and spatial resolution alongside biocompatibility must be considered for in vivo measurements in the brain [43]. Recently, E2 was characterized using fast-scan cyclic voltammetry (FSCV) [44] and co-detection with DA was achieved with a modified waveform [45] which satisfied the temporal and spatial requirements for both compounds.

Vertically aligned carbon nanofibers (VACNFs) have demonstrated excellent selectivity and sensitivity for DA sensing in the presence of common interferents such as ascorbic acid (AA) and uric acid (UA) [46], resistance to biofouling [41], and remarkable biocompatibility with neural cells [47]. In this study, we aim to explore VACNF as an electrode material for the direct electrochemical co-detection of E2 and DA.

## Materials and methods

### Electrode fabrication

**Fig. 2:**
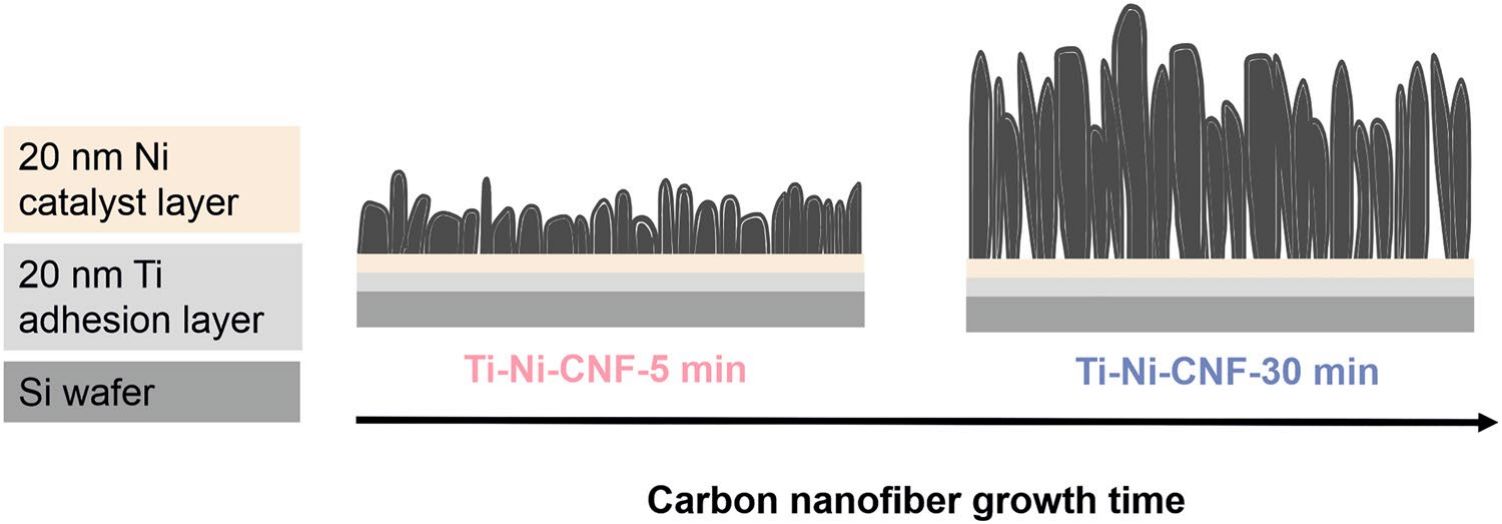
A schematic illustration of the preparation of Ti-Ni-CNF electrodes (dimensions are not to scale)

The electrode materials were fabricated as described in previous work [48]. The substrate is a p-type Si wafer (Siegert Wafers, Germany), first coated with a 20 nm Ti adhesion layer, followed by a 20 nm Ni catalyst layer. The two metal layers were deposited using an electron beam evaporator (MASA IM-9912) at roughly 2 × 10^−7^ mbar chamber pressure. Following this, the wafers were cleaved into smaller segments, measuring around 7 mm × 7 mm. The CNFs were grown via plasma-enhanced chemical vapor deposition (Aixtron Black Magic). Initially, the chamber was evacuated to 0.1 mbar. Subsequently, the chamber temperature was raised to 400 °C at a rate of around 250 °C per minute. Upon reaching 395 °C, 100 sccm NH_3_ buffer was introduced into the chamber. The ramp speed was then increased to 300 °C per minute, elevating the temperature to 600 °C. At 575 °C, a 230 W DC plasma was initiated, accompanied by the injection of 30 sccm C_2_H_2_ into the chamber, while the NH_3_ flow rate was raised to 125 sccm. These settings were upheld for durations of 5 and 30 min at 600 °C to prepare Ti-Ni-CNF-5 min and Ti-Ni-CNF-30 min, respectively. The chamber maintained a pressure of approximately 3 mbar throughout the growth process. The schematic illustration of the process is shown in Fig. 2.

To assemble the electrodes, a piece of sample was affixed onto a conductive copper sheet. Contact was ensured and enhanced by scratching the backside of the sample with a diamond pen and then copper. The geometric surface area of the electrode was defined by exposing a 3 mm diameter hole, while the remainder of the electrode was covered and sealed with polytetrafluoroethylene tape.

### Electrochemical measurements

The electrochemical measurements were performed using a Gamry Reference 620 (Warminster, PA, USA) potentiostat and a three-electrode configuration with an Ag/AgCl reference electrode, a Pt counter electrode, and the fabricated Ti-Ni-CNF working electrode. A phosphate buffer saline (PBS) solution was prepared by dissolving 80 g NaCl (> 99%, Sigma Aldrich), 2.0 g KCl (VWR), 14.4 g Na_2_HPO_4_ (Merck), and 2.4 g KH_2_PO_4_ (VWR) in 10 L of deionized water (resistivity 18.2 MΩ cm, Milli-Q, Millipore, Billerica MA) with a final pH of 7.4. The PBS solution was bubbled with N_2_ for at least 15 min, and then 50 mL was transferred onto the electrochemical cell with a stream of N_2_ above the solution kept throughout the experiment.

Cyclic voltammetry (CV) was performed using a potential window of 0–0.7 V and scan rates of 0.01–0.40 V/s. Prior to any measurement, multiple cycles were run in PBS pH 7.4 to stabilize the baseline reading. Afterwards, each CV run was set to three cycles. Differential pulse voltammetry (DPV) was done using the same potential window, with a step size of 0.01 V, sample period of 1 s, pulse time of 0.5 s, and pulse size of 0.10 V.

Stock solutions of 17-β-estradiol (> 99.9%), progesterone (> 99%), and testosterone (> 99.0%), all purchased from Sigma Aldrich, were prepared using absolute ethanol (> 99.5%, Altia). The solutions were stored in an amber glass vial covered with aluminium foil at 4 °C and were kept for use within 30 days. Dopamine solutions were prepared fresh on the day of the experiment by dissolving dopamine hydrochloride (Sigma Aldrich) in PBS. Different concentrations were achieved by the addition of the stock solution to the PBS in the cell and bubbling with N_2_ for at least 30 s to ensure thorough mixing. All measurements were conducted at room temperature.

### Data analysis

Calibration sensitivity, indicative of the electrode response to changes in E2 concentration, was obtained as the slope of the calibration curve, expressed in the unit µA/µM. The limit of detection (LOD) was calculated as follows: $LOD = 3\,sd/m$, where *sd* is the standard deviation of measurements done in PBS and *m* is the calibration sensitivity obtained from the calibration curve.

Electrochemical data was analysed using Gamry Echem Analyst 2. Data visualization and statistical analysis were done using MATLAB R2022a and Origin 2016 (Academic). Results are presented as mean ± standard deviation of three replicate measurements (n = 3). In the co-detection studies, paired t-test was used to compare results from the Ti-Ni-CNF-5 min and Ti-Ni-CNF-30 min electrodes, while one-way ANOVA was used to compare multiple measurements within each electrode type. All statistical tests were done at a 95% confidence level.

## Results and discussion

**Fig. 3:**
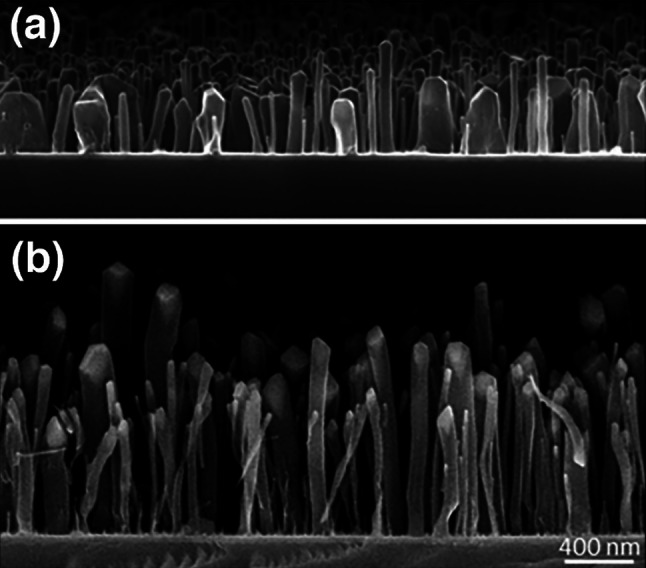
Cross-sectional SEM images of CNFs grown on Ti-Ni substrates for **a** 5 min and **b** 30 min, modified from [48] under the CC BY license

The CNFs have been extensively characterized previously [48–50]. Here, we outline the key characteristics observed in prior research, which have potential implications for interpreting the electrochemical findings presented in this paper. Table 1 summarizes the CNF dimensions obtained from scanning electron microscopy (SEM) images of 20 samples (Fig. 3) and some electrochemical properties. Remarkably, the longer growth time increased the average length of CNFs by 142%, whereas the average diameter of the CNFs was less affected.

**Table 1:** CNF specifications obtained from previous research

Electrode type	CNF length (nm)	CNF diameter (nm)	Analytical potential window in PBS pH 7.4 (mV)	Pseudocapacitance (µF/cm^2^)
Ti-Ni-CNF-5 min	361 ± 131	66 ± 51	1.43 ± 0.02	280 ± 22
Ti-Ni-CNF-30 min	873 ± 181	84 ± 43	1.23 ± 0.09	381 ± 19

Values are obtained from SEM images with sample size = 20 [50]

While conventional CNFs use Cr as an adhesive layer [51], replacing it with Ti resulted in wider analytical potential windows and smaller pseudocapacitance (C_dl_) [48]. As E2 oxidation is expected to occur at relatively high potentials, we investigate only Ti-Ni-CNF. Additionally, SEM images (Fig. 3) showed that this substitution decreases the CNF population density [48]. Moreover, a study on interfacial metal layer combinations demonstrated that Ni decreases the solubility of carbon and oxygen within the Ti-Ni system. As compared to Cr, this allowed the faster formation of segregated ordered carbon, and eventually of an electrochemically active surface layer [52]. The fabricated electrode materials contain trace amounts (1–3%) of Ti and Ni from the growth layer [49]. However, previous studies indicate that the electrochemical activity is primarily due to the carbon itself, with the metallic components at the interfaces playing a minor role [52].

Concerning the CNF growth time, it was observed that the Ti-Ni-CNF-30 min samples have a narrower potential window and higher C_dl_ [50], but the population density remains the same as Ti-Ni-CNF-5 min [48]. Previous experiments with X-ray photoelectron spectroscopy confirm that the chemical composition is similar, and no additional unexpected elements are found [48]. The rough electroactive surface area estimation based on the measured C_dl_ shows that the Ti-Ni-CNF-30 min samples have approximately 36% larger electroactive surface area than Ti-Ni-CNF-5 min, but show chemically identical characteristics [50]. The geometry of carbon nanostructures was found to influence the thin-layer and diffusion phenomenon [53] and for CNFs, longer fibers demonstrated some extent of thin-layer formation at medium scan rates [50].

The main advantages of the vertically aligned carbon nanofibers (VACNFs) are their excellent selectivity and sensitivity for DA sensing [46], resistance to biofouling [41], and remarkable biocompatibility with neural cells [47]. Our previous study demonstrates that merely by controlling the length of the CNFs, we can regulate the peak separation between AA, UA, and DA oxidation, thus the selectivity. These reports highlight the relationship between electrode composition, CNF morphology, and electrochemical behaviour. Different approaches in fabrication affect the fiber nucleation and can be controlled to obtain the desired structures for various applications.

### Electrochemical characterization of E2 oxidation

**Fig. 4:**
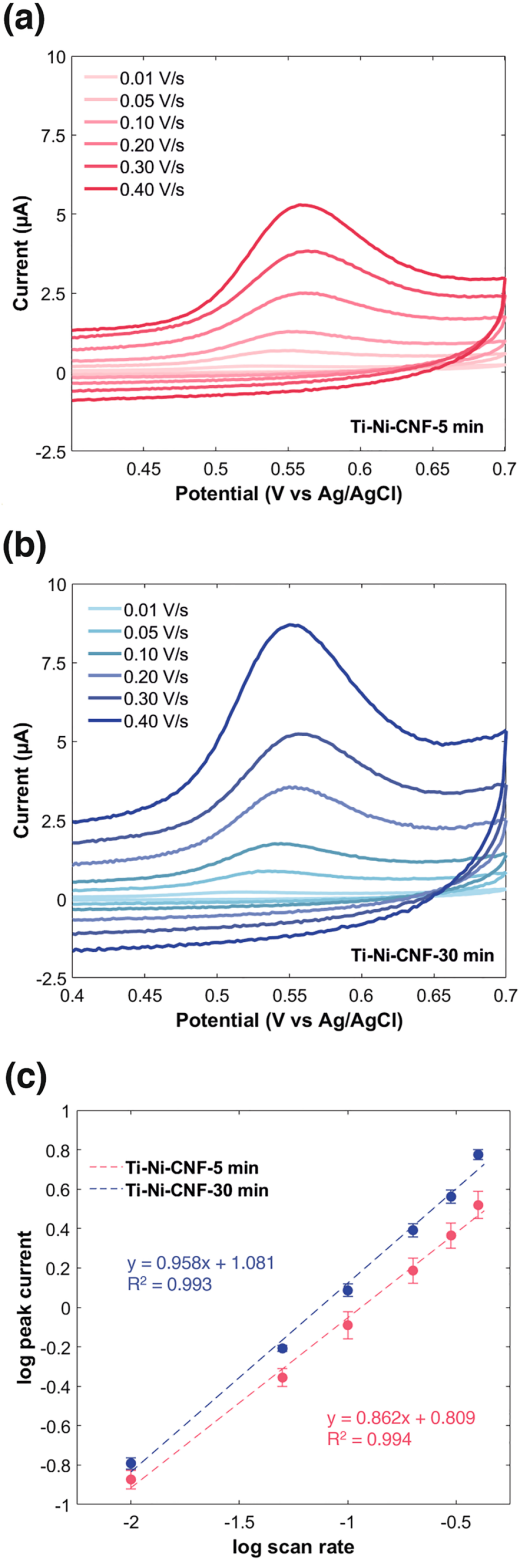
Cyclic voltammograms showing the oxidation of 10 µM E2 at different scan rates in **a** Ti-Ni-CNF-5 min and **b** Ti-Ni-CNF-30 min electrodes; **c** logarithmic plots of peak current versus scan rate. Measurements were done in 0.1 M PBS pH 7.4. Results are presented as mean ± standard deviation (error bars), where n = 3

To gain insights into the electrochemical behavior of E2 at the electrode surface, its oxidation was examined using CV at different scan rates. As depicted in Fig. 4a and b, E2 undergoes irreversible oxidation with an anodic peak potential (E_pa_) of about 0.55 V—characteristic of carbon-based electrodes. The average CNF length does not have a substantial impact on E_pa_, but as the scan rate increases, the E_pa_ values shift towards more positive potentials (Table S1).

As expected, Ti-Ni-CNF-30 min have higher faradaic and capacitive current values. The increase in CNF growth time not only enhanced the number of available sites for oxidation resulting in higher anodic peak current (I_pa_) but also increased the C_dl_ of the material [50]. Figure 4c combines the logarithmic plots of oxidation current *versus* scan rate in the two electrodes. A slope between 0.2 and 0.6 indicates a diffusion-controlled process, 0.75–1 implies adsorption-control, while values between 0.6 and 0.75 suggest a mixture of both [54]. Slopes close to 1 are also the expected value for pure-thin-layer behaviour, which is the phenomenon where the analyte is confined within the nanostructure. Due to their geometrical nature, the CNFs express (modest) thin-liquid-layer effects [50].

From the logarithmic plots in Fig. 4c, slopes equal to 0.862 and 0.958 were obtained for Ti-Ni-CNF-5 min and Ti-Ni-CNF-30 min, respectively. Both values imply adsorption-controlled kinetics. While most electrochemical characterization studies of E2 lean towards adsorption control [38, 44, 55], some reported diffusion control [35, 54] and both [36]. A slightly stronger influence of adsorption was observed in longer CNFs, with the slope in Fig. 4c being almost equal to 1. In DA detection with similar VACNF electrodes, enhanced adsorption was also observed with increasing average CNF length [46]. The adsorption of the analyte to the electrode material is a requirement in sensing both E2 and DA. However, the kinetics can be either diffusion- or adsorption-controlled. In this case, where adsorption is non-negligible, it can be inferred that the electrochemical behaviour is caused by the combination of an increase in the number of adsorption sites from the electroactive surface area as well as thin-layer formation, especially for longer CNFs.

### Linear range and sensitivity of E2 detection

**Fig. 5:**
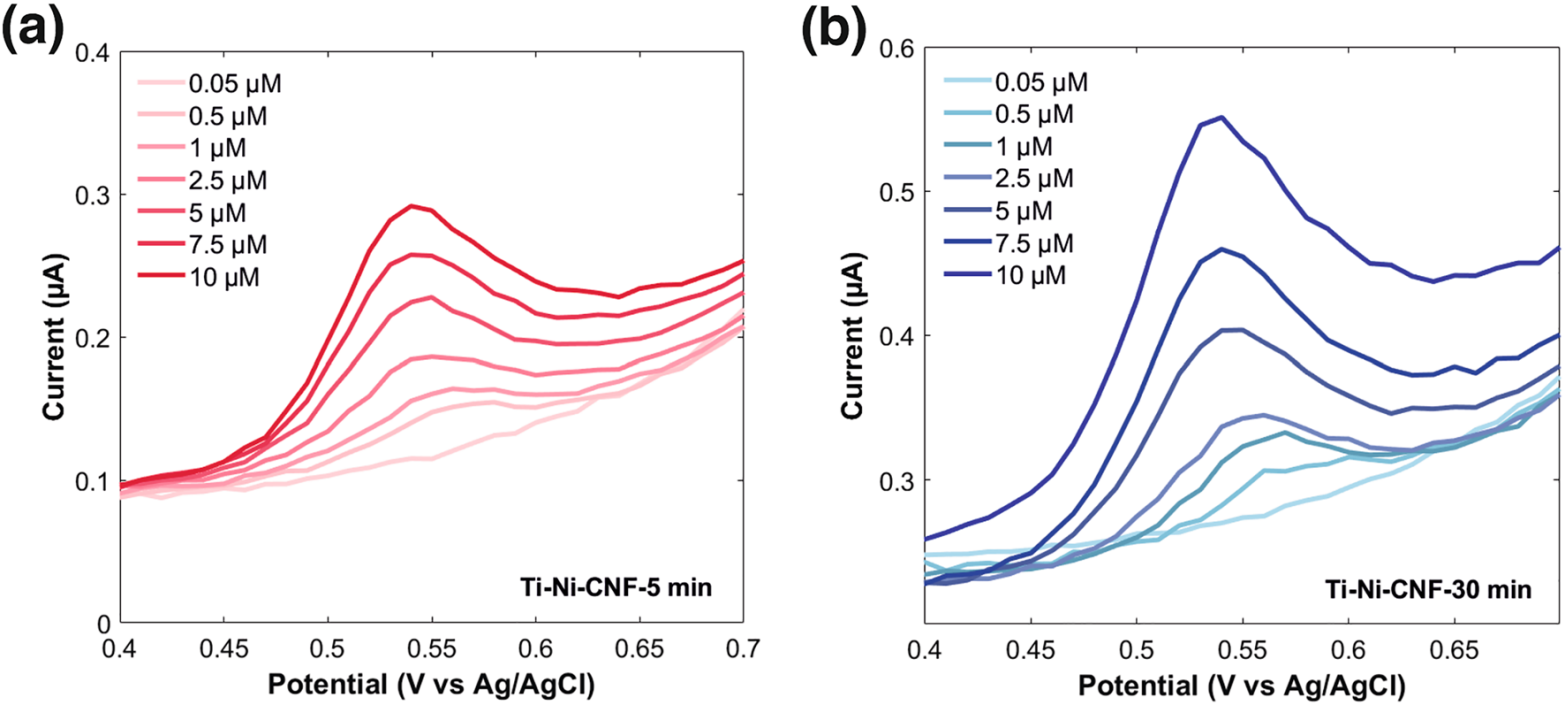
DPV responses of E2 concentrations ranging from 0.05 to 10 µM in 0.1 M PBS pH 7.4 at 0.1 V pulse amplitude in representative **a** Ti-Ni-CNF-5 min and **b** Ti-Ni-CNF-30 min electrodes

The performance of the CNF electrodes in sensing different E2 concentrations was assessed using DPV (Fig. 5). One advantage of this differential method is the lower contribution of the background capacitive current, thereby improving its sensitivity [56]. Consequently, the current readings obtained are smaller compared to CV, but the current values got larger with increased average CNF length. Both electrode types demonstrated a wide linear range of 0.05–10 µM, aligning closely with recent direct electrochemical E2 sensing involving carbon materials, as presented in Table 2. With the Ti-Ni-CNF electrodes, it could be possible to extend this range, but concentrations higher than 10 µM were intentionally omitted considering the useful values for the intended application.

**Table 2:** Comparison of performance characteristics of recent studies on direct E2 detection

Electrode	Electrochemical technique	Linear range (µM)	Calibration sensitivity (µA/µM)	Detection limit (nM)	References
PANI/CDs/GCE*	LSV	0.001–100	3.3	43	[38]
P(L-tyr)/AuNCs/PDACNTs/GCE*	DPV	0.05–10	1.72	7.1	[39]
wMC0.67/GCE*	DPV	A. 0.05–10, B. 10–80	A. 0.341, B. 0.0620	8.3	[55]
rGO-AuNPs/CNT/SPCE	DPV	0.05–1	0.583	3	[35]
CFME	FSCV	0.1–10	0.00571	31.2	[44]
Ti-Ni-CNF*	DPV	0.05–10	5 min: 0.016 ± 0.001 30 min: 0.020 ± 0.004	5 min: 154 30 min: 207	This work

*PANI* polyaniline, *CD* carbon dot, *GCE* glassy carbon electrode, *tyr* tyrosine, *AuNC* gold nanocluster, *PDACNT* polydopamine modified carbon nanotube, *wMC* wrinkled mesoporous carbon, *rGO* reduced graphene oxide, *AuNP* gold nanoparticle, *SPCE* screen-printed carbon electrode, *CFME* carbon fiber microelectrode, *LSV* linear sweep voltammetry

Electrodes marked with * have the same geometric area = 0.0071 cm^2^

**Fig. 6:**
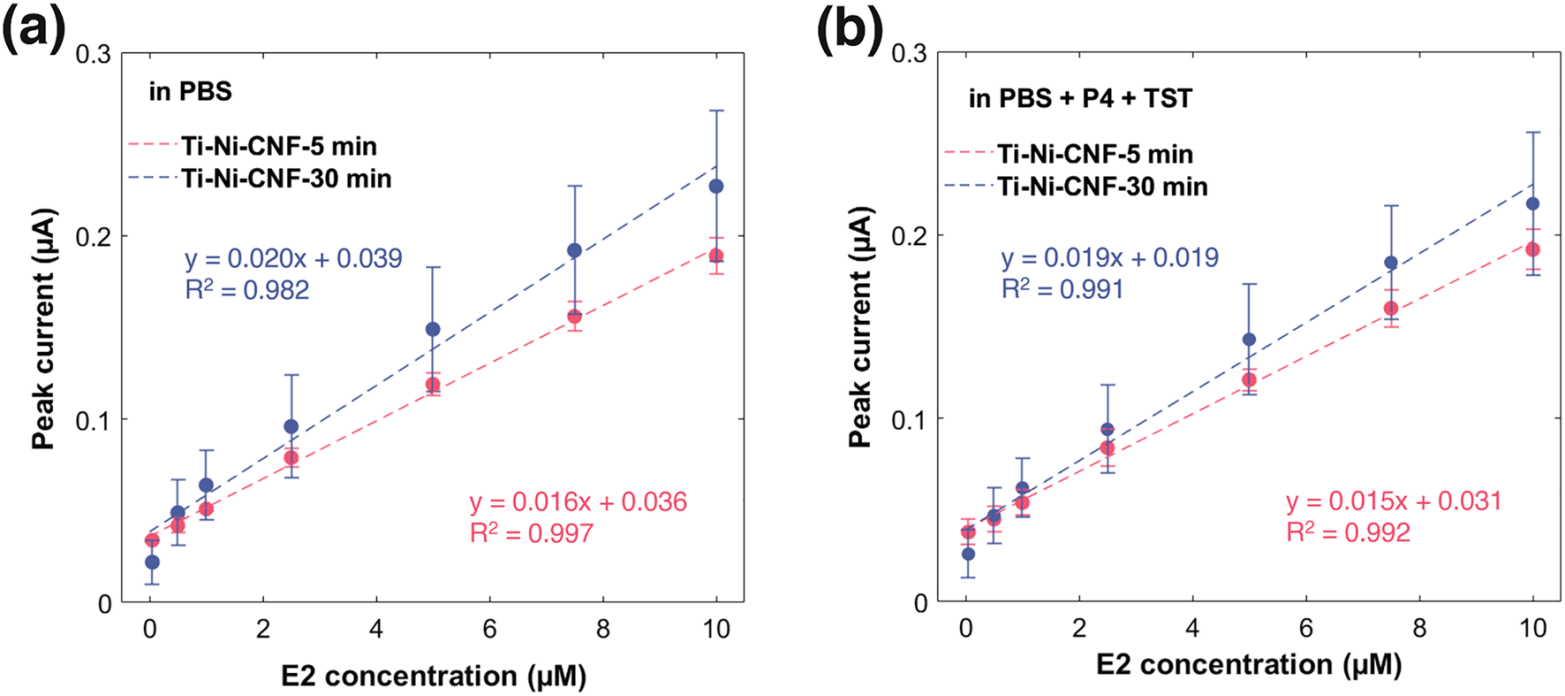
Calibration curve of E2 in Ti-Ni-CNF electrodes in **a** PBS pH 7.4 and **b** PBS pH 7.4 + 0.065 µM P4 + 0.0025 µM TST. Results are presented as mean ± standard deviation (error bars), where n = 3

A slight increase in sensitivity was obtained, from 0.016 µA/µM in Ti-Ni-CNF-5 min to 0.020 µA/µM in Ti-Ni-CNF-30 min. Looking at Fig. 6a, the distinction between the average fiber lengths is less pronounced at lower E2 concentrations, where a larger available surface area does not offer any advantage. Then, the regression lines diverge as the amount of E2 increases, particularly the one associated with Ti-Ni-CNF-5 min becoming less steep. Thus, the effect of average CNF length becomes more prominent at higher E2 concentrations, with Ti-Ni-CNF-30 min providing a greater number of available adsorption sites for E2.

### Limit of detection

The variability in fiber length, primarily attributed to the stochastic nature of the growth process, is more prominent with the extended growth time for CNFs. Consequently, this has influenced the repeatability of results obtained from Ti-Ni-CNF-30 min electrodes throughout this study. Increased deviations were observed between measurements in the blank PBS at pH 7.4. As a result, the Ti-Ni-CNF-30 min electrodes exhibited a high LOD of 207 nM, whereas Ti-Ni-CNF-5 min electrodes had a slightly lower value of 154 nM (Table 2). Similar observations were reported in previous studies using Cr-Ni-CNF electrodes in PBS [46]. With the use of DPV, a technique with a lower contribution from the capacitive current, smaller LODs were calculated in this work.

To effectively measure hormone fluctuations during the various stages of the menstrual cycle, sensors with lower LODs, ideally reaching picomolar levels, are needed. This has not been achieved using any direct electrochemical sensing studies. However, sensors with LODs in the middle to high nanomolar range can still be useful. In a study monitoring hormone concentration in uncomplicated pregnancies, estradiol levels were found to reach up to 80 nM in the third trimester for primiparous women [57]. Nevertheless, factors such as biofouling are still to be considered.

CNFs generally have a vertical, forest-like morphology. For Ti-Ni-CNFs, transmission electron microscopy (TEM) micrographs (Fig. S1c and d) revealed a relatively disordered structure with exposed basal planes and rich surface chemistry, providing adsorption sites for different functional groups [49]. While defects in the microstructure enhance chemistry, finding its balance with highly controlled fiber growth is crucial for attaining more stable baselines, enhancing the repeatability of measurements, and lowering LODs.

### E2 detection in the presence of other sex hormones

The performance of CNF electrodes in E2 detection was assessed in the presence of two other sex hormones, P4 and TST. The solutions were prepared in PBS pH 7.4, with the hormone concentrations reflecting the maximum physiological levels found in blood serum. While serum concentrations can vary depending on reproductive phase and lifestyle factors, premenopausal serum P4 has been reported to reach up to 0.050–0.065 µM [58–60] with a corresponding E_pa_ of about 0.8 V [61]. Serum TST in premenopausal females has been reported to reach approximately 0.0025 µM [62, 63] with a peak reduction potential of − 1.4 V [64].

With the mentioned peak potentials, it is expected that the two other sex hormones will not significantly interfere with E2 detection. However, considering the structural similarities among the three hormones, particularly the hydroxyl (–OH) or carbonyl (–C=O) group attached to the sterol nucleus (Fig. 1), it is still interesting to know whether the presence of P4 and TST affects the electrochemical sensing of E2. In the presence of P4 and TST, the E_pa_ of E2 remained at around 0.55 V (Table S2), and no additional peaks were observed. While a minor decrease in I_pa_ was noted across various E2 concentrations, Fig. 6b shows that the linear range of 0.05–10 µM is preserved. However, a slight decrease in sensitivity was observed compared to E2 detection in the absence of interfering hormones. The slopes of the calibration curve decreased by 0.001 µA/µM in both types of Ti-Ni-CNF electrodes.

### Co-detection with dopamine

Our VACNF electrodes have been successfully used for the detection of DA [46] and E2 (this work). In this section, we explored the co-detection of E2 and DA using DPV. To the best of our knowledge, there is only one other reported co-detection study of E2 and DA, which involved electrochemical waveform modification to achieve good peak separation and sensitivity for the two analytes [45]. Here, we are interested in the effect of material modification to E2 and DA sensing.

Both E2 and DA act as inner-sphere molecules that require direct adsorption to the electrode surface for oxidation to occur. Calculations have shown that there is a strong non-covalent π-π interaction between the aromatic ring of DA and the basal sites in the annealed graphene surface model [65]. Similarly, theoretical studies described the physical adsorption of E2 and graphene to be primarily attributed to π-π interactions [66]. TEM micrographs from previous works (Fig. S1c and d) show that the Ti-Ni-CNF electrodes are made up of basal graphene sheets. In this work, these basal planes are referred to as adsorption sites for E2 and DA.

**Fig. 7:**
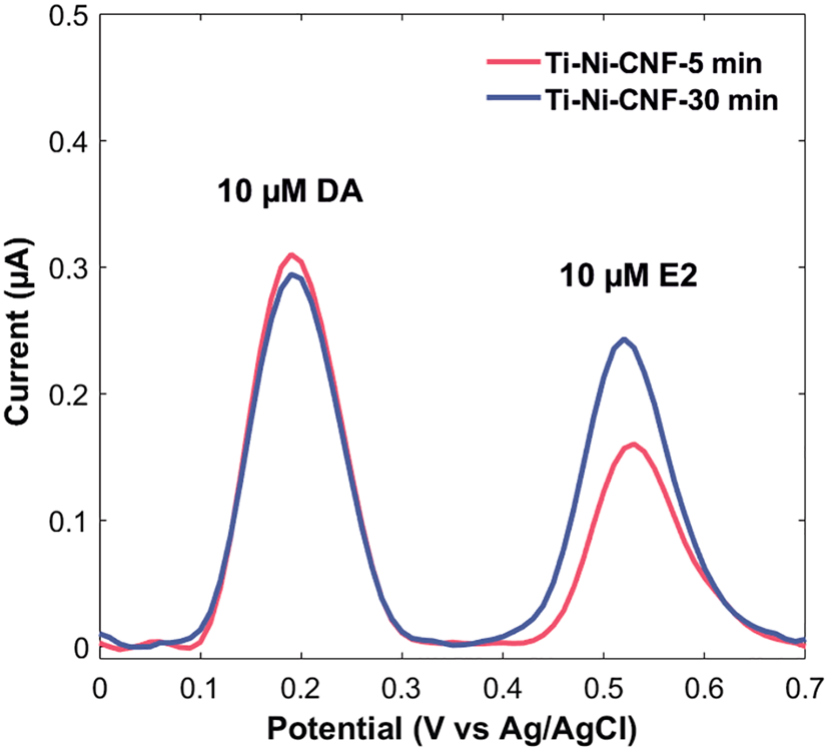
Average DPV responses of 10 µM E2 and DA in 0.1 M PBS pH 7.4 at 0.1 V pulse amplitude in Ti-Ni-CNF electrodes, where n = 3

As the E_pa_ of DA is lower than E2, it is oxidized first. The two peaks are well resolved and the peak positions are consistent with literature, around 0.20 V for DA and 0.55 V for E2. However, a drifting baseline was observed towards the E2 peak, as expected due to its position towards the end of the potential window (Fig. S2). The combined voltammograms from the two electrode types upon baseline correction is shown in Fig. 7. The CNF growth time had minimal influence on peak separation (ΔE): 0.34 V ± 0.01 and 0.33 V ± 0.01 for Ti-Ni-CNF-5 min and Ti-Ni-CNF-30 min electrodes, respectively. This agrees with the ΔE for E2 and DA obtained using an unmodified waveform for FSCV [45].

When the two compounds are present, each containing one aromatic ring (Fig. 1), there is competition for available adsorption sites for electrochemical detection. Because of its lower E_pa_, the competitive adsorption of DA to contributed in the decreased E2 oxidation current for both electrode types, reflected in the smaller E2 DPV peaks in Fig. 7. Through paired samples *t*-test at a 95% confidence level, it was discovered that the E2 and DA peaks in Ti-Ni-CNF-5 min electrodes differ significantly, while the same statistical analysis revealed no significant difference in the two peaks found in Ti-Ni-CNF-30 min electrodes (Figs. 7 and S2).

The electrode response to equal concentrations of E2 and DA is expressed as the E2/DA peak current ratio, which is expected to be close to one when the material has equal sensitivity to both compounds. One approach to achieve this is the optimisation of electrochemical technique, such as waveform modification in FSCV, improving ΔE and the E2/DA peak current ratio from 0.39 ± 0.07 to 0.75 ± 0.07 [45]. Our work focuses on a material-driven approach. If the available electrochemically active surface sites for adsorption is limited, the sensitivity of detection decreases. We consider adsorption sites to be abundant when competitive adsorption does not limit sensitivity. Here, we obtained a ratio of 0.53 ± 0.07 for Ti-Ni-CNF-5 min and 0.81 ± 0.16 for Ti-Ni-CNF-30 min. A 53% increase was achieved by regulating the CNF growth time during material fabrication. Indeed, in co-detection scenarios where both analytes rely on adsorption for oxidation, the availability of adsorption sites emerges as a critical factor for effective electrochemical detection.

**Fig. 8:**
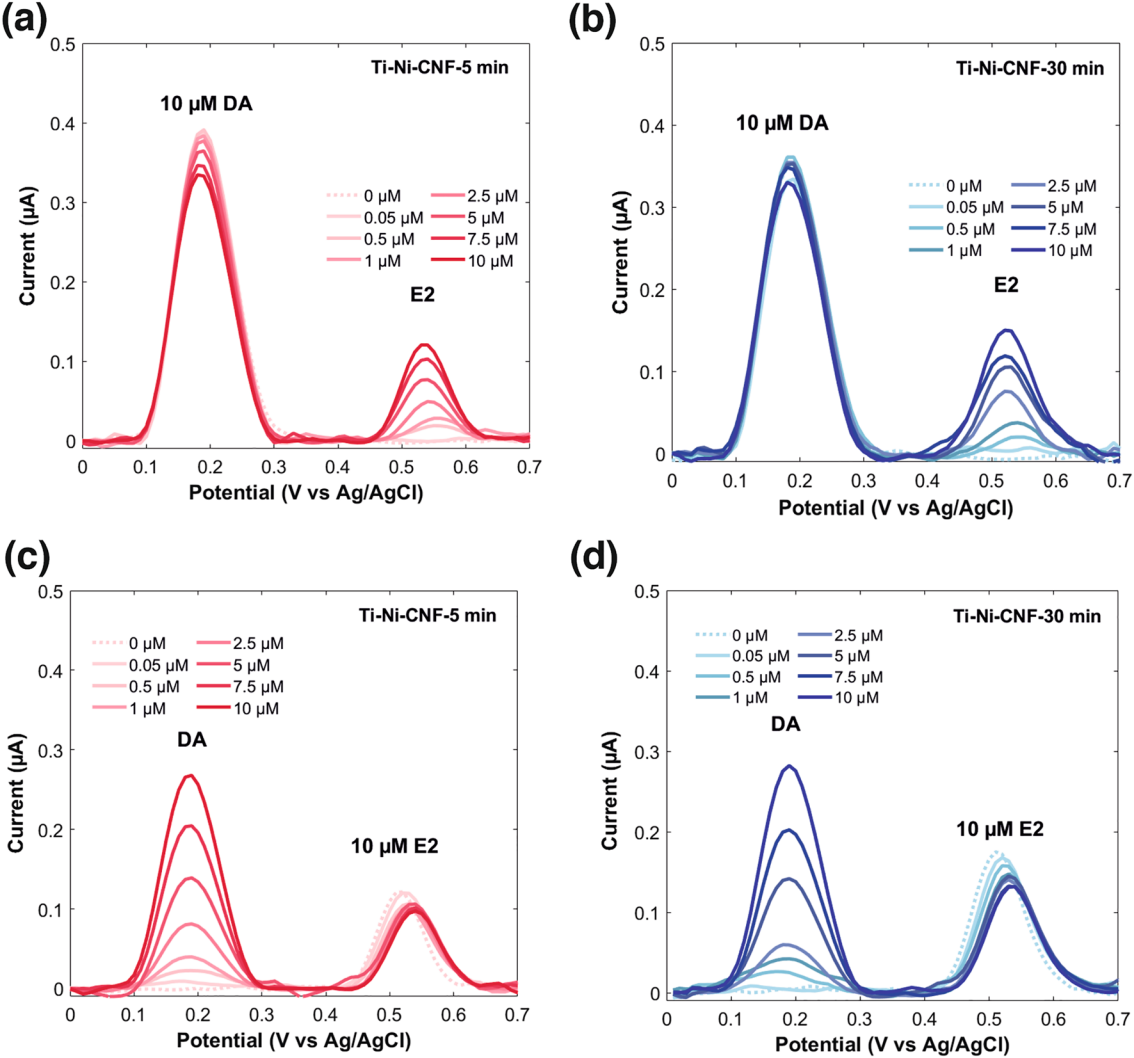
DPV responses of 10 µM DA and 0.05–10 µM E2 in Ti-Ni-CNF-5 min (**a**), and Ti-Ni-CNF-30 min electrodes (**b**); and 10 µM E2 and 0.05–10 µM DA in Ti-Ni-CNF-5 min (**c**), and Ti-Ni-CNF-30 min electrodes (**d**), respectively. All measurements were done in 0.1 M PBS pH 7.4 at 0.1 V pulse amplitude

To further investigate the co-detection of E2 and DA, 0.05–10 µM of each analyte were measured in the presence of high concentration (10 µM) of the other, as depicted in Fig. 8. E2 and DA peaks in all the cases remained well-separated, and E2/DA current peak ratios at 10 µM concentrations of both compounds were calculated to be: 0.36 ± 0.05, 0.47 ± 0.02, 0.40 ± 0.03, and 0.48 ± 0.06, respectively. In brief, lower ratios were obtained in all scenarios compared to the straightforward equimolar E2 and DA measurements. Here, we discuss the influence of adsorption sites in more detail, considering the average lengths of the CNFs and the oxidation potentials of E2 and DA.

A constant and high DA concentration led to decreased sensitivity and linearity of the E2 measurements in both electrode types (Fig. S3a) compared to measurements done in PBS alone (Fig. 6). Given the nature of the two analytes and the predicted competition for adsorption sites, DA is expected to overwhelm E2, particularly at lower concentrations. The DA peaks in Fig. 8a and b appear stable at consistent peak potentials, although there is a noticeable decrease in peak currents along the multiple DPV runs.

Remarkably demonstrating the influence of adsorption sites in analyte detection, measurements performed using Ti-Ni-CNF-5 min gave the lowest E2/DA peak current ratio and sensitivity; the lowest E2 concentration (0.05 µM) was not detected. The multiple DPV runs at a high DA concentration lowered the number of available adsorption sites, leading to smaller sensitivity and shortened linear range. Interestingly, a higher sensitivity (Fig. S3a) was obtained with the Ti-Ni-CNF-30 min electrodes under the same conditions, and the lowest E2 concentration was detected. With a greater number of electrochemically active sites, the E2/DA peak current ratio also increased by 30%.

In comparison to DA, a high and constant E2 concentration showed more pronounced changes in current peak height and peak position as the measurement progressed (Fig. 8c and d). Notably, E2 did not interfere with the measurement of increasing DA concentrations, which exhibited excellent linear relationship and sensitivity (Fig. S3b). Among the electrodes, the Ti-Ni-CNF-30 electrodes gave the best values, clearly demonstrating the key role of adsorption site abundance in sensor performance.

**Fig. 9:**
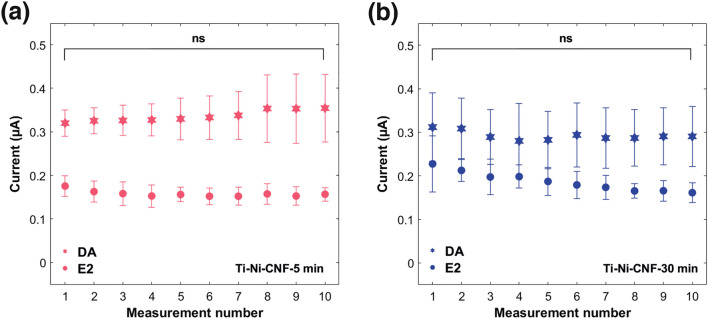
Current responses of the 10 repeated measurements in **a** Ti-Ni-CNF-5 min and **b** Ti-Ni-CNF-30 min (one-way ANOVA at a 95% confidence level, n = 3, ns = no significant difference). Results are presented as mean ± standard deviation (error bars), where n = 3

Nine additional DPV measurements were made using the same concentrations of E2 and DA to assess the repeatability of the current responses. Figure 9 illustrates stable readings obtained for both compounds across all 10 measurements. Using one-way ANOVA at a 95% confidence level, no significant differences were observed among the 10 measurements for both E2 and DA signals in both Ti-Ni-CNF electrode types.

Long-term stability is another important parameter for continuous measurements, but this entails the property not only of the electrode material but also of the fabricated sensor as a whole. A simple measurement was performed to check the performance of Ti-Ni-CNF electrodes after five days (Fig. S4). It is difficult to obtain substantial conclusions from the experiment, but some of the results are discussed in the Supplementary note 2.

## Conclusions

Our study has successfully demonstrated the direct electrochemical co-detection of E2 and DA using Ti-Ni-CNF electrodes. The VACNFs had been previously studied for DA sensing, so this work started with electrochemical characterisation of E2. Using CV, E2 oxidation was described as an irreversible and adsorption-controlled process. In employing a more sensitive technique such as DPV, we achieved a wide linear range and satisfactory sensitivity even in the presence of other steroid hormones such as P4 and TST. More specifically, the sensitivity of E2 detection in PBS improved from 0.016 µA/µM in Ti-Ni-CNF-5 min electrodes to 0.020 µA/µM in Ti-Ni-CNF-30 min.

Co-detection experiments on E2 and DA showed well-resolved peaks with ΔE of 0.30 ± 0.01 V and repeatability after 10 measurements. In detecting equimolar E2 and DA, the E2/DA peak current ratio increased from 0.53 ± 0.07 to 0.81 ± 0.16 with longer CNF growth time. The 53% improvement can be attributed to the increase in average CNF length and the calculated 36% larger electroactive surface area. Additional investigations, using a constant concentration of one analyte and increasing concentrations of the other, further supported this conclusion. The Ti-Ni-CNF-30 min electrodes exhibited the best performance for co-detection in terms of peak current ratios, sensitivity, and linearity. These findings highlight the role of adsorption site abundance in co-detection of E2 and DA. The oxidation of both compounds require adsorption to the electrode surface, which occurs through the same π-π interaction in the aromatic ring in each molecule and the basal graphene sheets in the CNF structures. With the competitive adsorption between E2 and DA, having numerous electroactive sites that can be regulated during material fabrication is beneficial in achieving desired sensitivity.

The unique mechanical properties of CNFs, such as selectivity towards DA in the presence of known interferences such as AA and UA, reduced fouling, and biocompatibility with neural cells, make them promising materials for sensing in the brain. The development of multianalyte sensing platforms can enable further investigations into the role of E2 as a neuromodulator of DA, and its broader impact, along with other female hormones, on brain functions. Improving fiber growth to minimise variations across the electrode surface is important in achieving lower LODs. Looking ahead, research efforts should aim to enhance the co-detection capabilities at low and fluctuating analyte concentrations and address spatial and temporal requirements for E2 and DA.

### Supplementary Information

Additional file1 (DOCX 2936 kb)

## Data Availability

The authors declare that the data supporting the findings of this study are available within the paper. Data sets generated during the current study are available from the corresponding author on reasonable request.
