# Supplementary material for: Key role of adsorption site abundance in the direct electrochemical co-detection of estradiol and dopamine
Source: Discov Nano. 2024 Aug 28;19(1):134. doi: 10.1186/s11671-024-04092-8 (PMC11358574; doi:10.1186/s11671-024-04092-8)
Supplement: Supplementary file 1 — Additional file1 (DOCX 2936 kb) [file 11671_2024_4092_MOESM1_ESM.docx]

**Key Role of Adsorption Site Abundance in the Direct Electrochemical Co-Detection of Estradiol and Dopamine**

Naela Delmo^1^, Ishan Pande^2^, Emilia Peltola^1,2^

^1^ Department of Mechanical and Materials Engineering, Faculty of Technology, University of Turku, 20500 Turku, Finland

^2^ Department of Electrical Engineering and Automation, School of Electrical Engineering, Aalto University, 00076 Aalto, Finland

**Supplementary Information**

**Supplementary tables**

**Table S1** Anodic peak potential (E_pa_) of E2 in PBS pH 7.4 in different scan rates using CV (potential window 0 – 0.7 V)

| **Scan rate (V/s)** | **E_pa_ (V)** | |
| --- | --- | --- |
|  | **Ti-Ni-CNF 5 min** | **Ti-Ni-CNF 30 min** |
| 0.400 | 0.555 ± 0.003 | 0.566 ± 0.008 |
| 0.300 | 0.557 ± 0.001 | 0.567 ± 0.006 |
| 0.200 | 0.552 ± 0.002 | 0.565 ± 0.002 |
| 0.100 | 0.545 ± 0.001 | 0.554 ± 0.004 |
| 0.050 | 0.536 ± 0.002 | 0.543 ± 0.004 |
| 0.010 | 0.517 ± 0.005 | 0.530 ± 0.002 |

**Table S2** Anodic peak potential (E_pa_) of E2 in PBS pH 7.4 and in the presence of 0.065 µM P4 and 0.0025 µM TST using DPV (potential window = 0 – 0.7 V, step size = 0.01 V, sample period = 1 s, pulse time = 0.5 s, and pulse size = 0.10 V)

| **E2 concentration (µM)** | **E_pa_ (V)** | | | |
| --- | --- | --- | --- | --- |
|  | **Ti-Ni-CNF 5 min** | | **Ti-Ni-CNF 30 min** | |
|  | In PBS pH 7.4 | In PBS pH 7.4 + 0.065 µM P4 + 0.0025 µM TST | In PBS pH 7.4 | In PBS pH 7.4 + 0.065 µM P4 + 0.0025 µM TST |
| 0.05 | 0.597 ± 0.012 | 0.575 ± 0.007 | 0.580 ± 0.017 | 0.597 ± 0.015 |
| 0.5 | 0.560 ± 0.010 | 0.577 ± 0.015 | 0.570 ± 0.010 | 0.587 ± 0.015 |
| 1 | 0.560 ± 0.000 | 0.563 ± 0.006 | 0.560 ± 0.000 | 0.577 ± 0.006 |
| 2.5 | 0.543 ± 0.012 | 0.566 ± 0.029 | 0.547 ± 0.006 | 0.557 ± 0.006 |
| 5 | 0.540 ± 0.000 | 0.546 ± 0.006 | 0.537 ± 0.006 | 0.540 ± 0.000 |
| 7.5 | 0.540 ± 0.000 | 0.540 ± 0.000 | 0.540 ± 0.000 | 0.551 ± 0.028 |
| 10 | 0.540 ± 0.000 | 0.543 ± 0.006 | 0.530 ± 0.000 | 0.540 ± 0.000 |

**Supplementary Note 1**

For DA measurements, selectivity towards both ascorbic acid and uric acid is relevant in certain settings. Our previous efforts were concentrated on detecting DA, with confirmed selectivity towards both ascorbic acid and uric acid using Cr-Ni-CNFs [46]. For this study, the Ti-Ni-CNFs were selected due to their wider potential window necessary for E2 detection. Moreover, unpublished results confirm that the 30-minute grown Ti-Ni-CNFs show similar selectivity to that of 30-minute grown Cr-Ni CNFs [Personal discussion with Ayesha Kousar]. Ti-Ni-CNFs grown for 5 and 10 minutes did not exhibit selectivity, most likely due to their lesser surface population densities in comparison to Cr-Ni-CNFs. On the other hand, ascorbic acid does not necessarily interfere with the electrochemical detection of DA [doi.org/10.1038/s41598-022-24580-0]. We wanted to include Ti-Ni-CNF-5 min to current study as it has wider potential window than Ti-Ni-CNF-30 min.

**Supplementary note 2**

The electrodes used in the previous experiments were stored in 0.1 M PBS pH 7.4 at 4°C. After the stated incubation period, measurements were done both in PBS alone and in 10 µM of both E2 and DA. Some of the electrodes have recovered well and did not show any residual peaks in PBS. Some were able to detect the two analytes, although the change in peak heights are inconsistent among the electrodes and seems to be not related to either the oxidation potential or average lengths of the CNFs.

Furthermore, some of the electrodes were damaged, possibly due to the buffer seeping into the insulating cover. Our sample holder is not designed for long term experiments and the polytetrafluoroethylene tape easily leaks. It is particularly challenging to obtain good long-term attachment for the tape for the highly 3-dimensional structure. When the tape holds, stability appears good as demonstrated by the rest of the experiments in the manuscript. Fig S4 shows an example where lower peak currents were observed for both E2 and DA after five days.

**Supplementary figures**


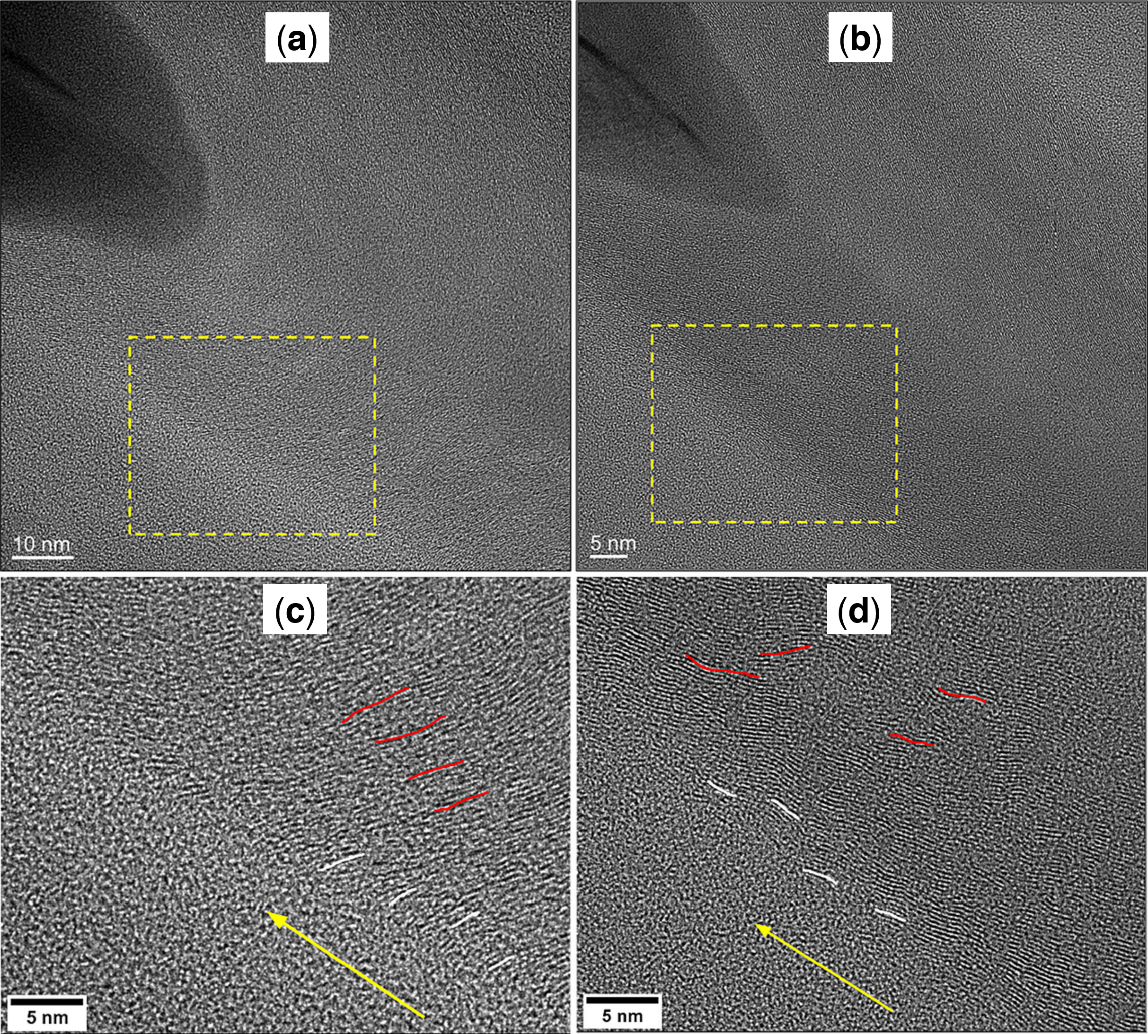


**Fig. S1** High magnification TEM images of **a, c** CrNi-CNF and **b, d** TiNi-CNF. The yellow rectangles in **a** and **b** indicate the sections of the figures which are shown in **c** and **d**, respectively. The brightness and contrast were adjusted in **c** and **d** to clearly show the graphene sheets. Graphene sheets close to the fiber edge are traced with white lines, while graphene sheets that are further inside the fiber are traced with red lines. The yellow arrows in **c** and **d** indicate the direction of the fiber's axis. Reproduced from [49] under the CC BY license


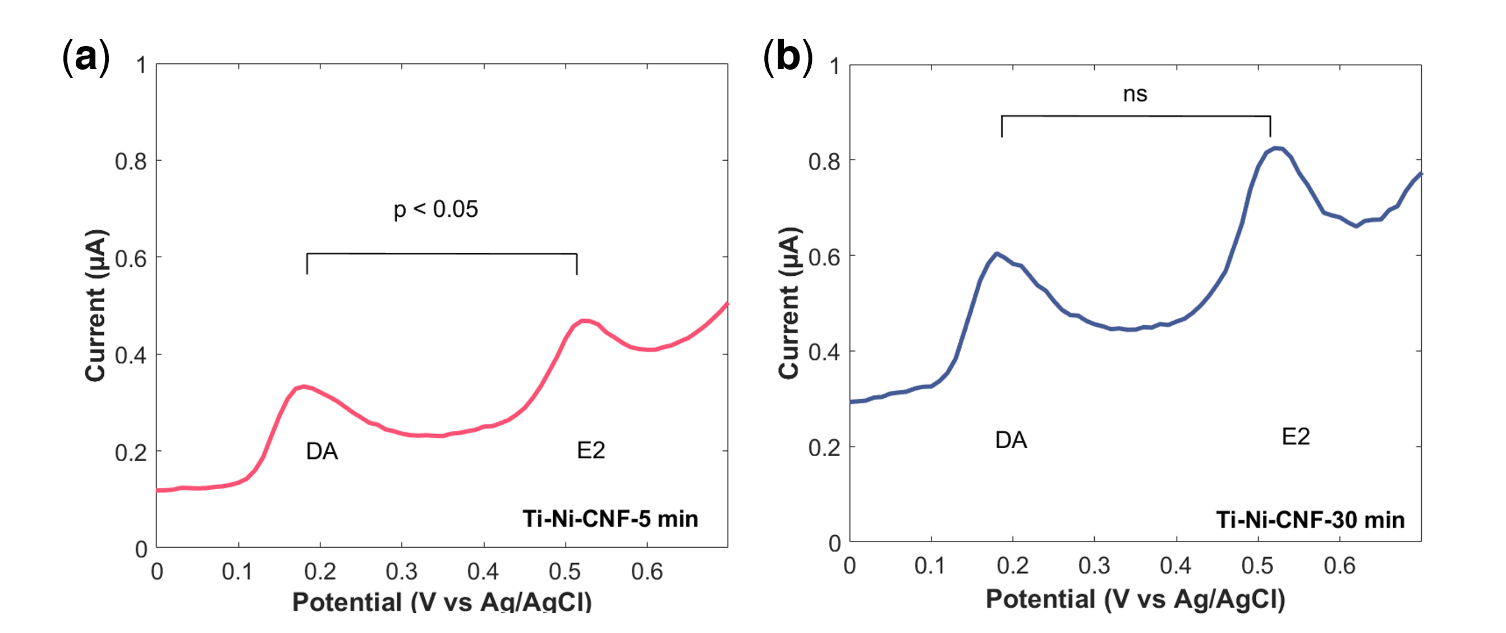


**Fig. S2** DPV response of 10 µM DA and E2 in PBS pH 7.4 at 0.1 V pulse amplitude in representative **a** Ti-Ni-CNF-5 min and **b** Ti-Ni-CNF-30 min electrodes (paired t-test at a 95 % confidence level, n = 3, ns = no significant difference)


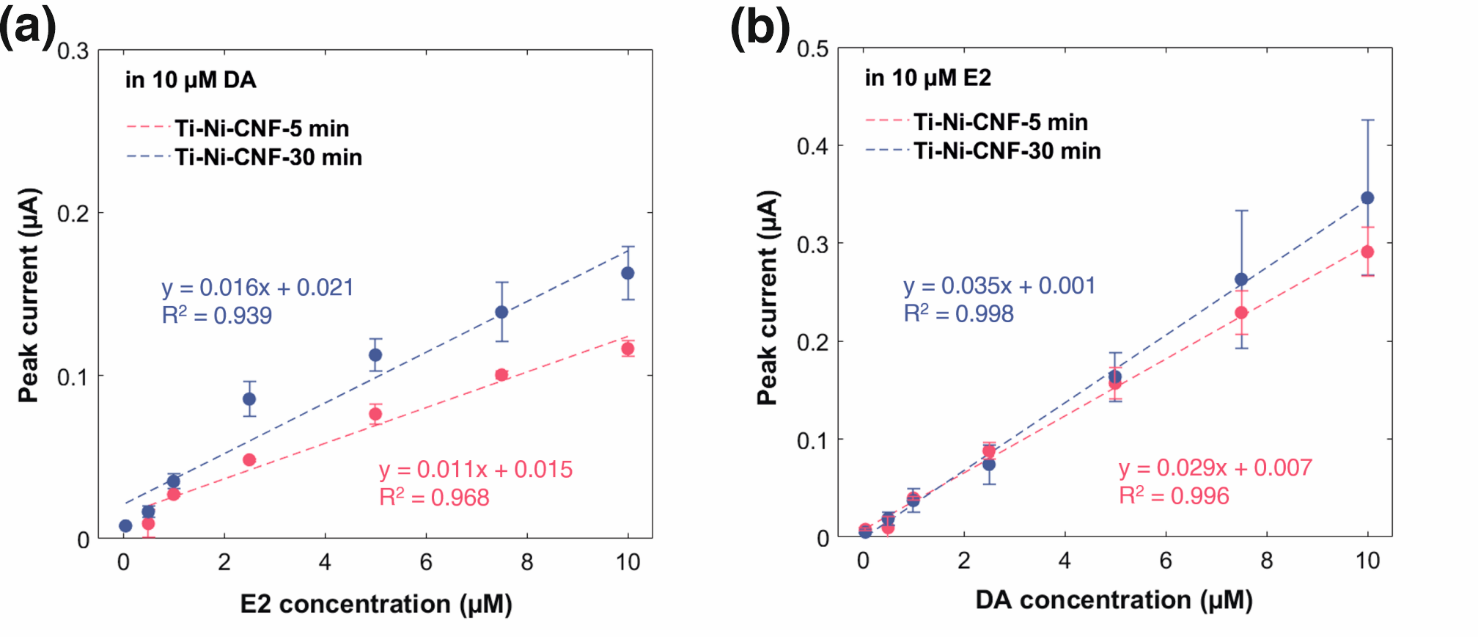


**Fig. S3** Calibration curves of **a** E2 in 10 µM DA and **b** DA in 10 µM E2 from DPV responses using Ti-Ni-CNF electrodes. Results are presented as mean ± standard deviation (error bars), where n = 3


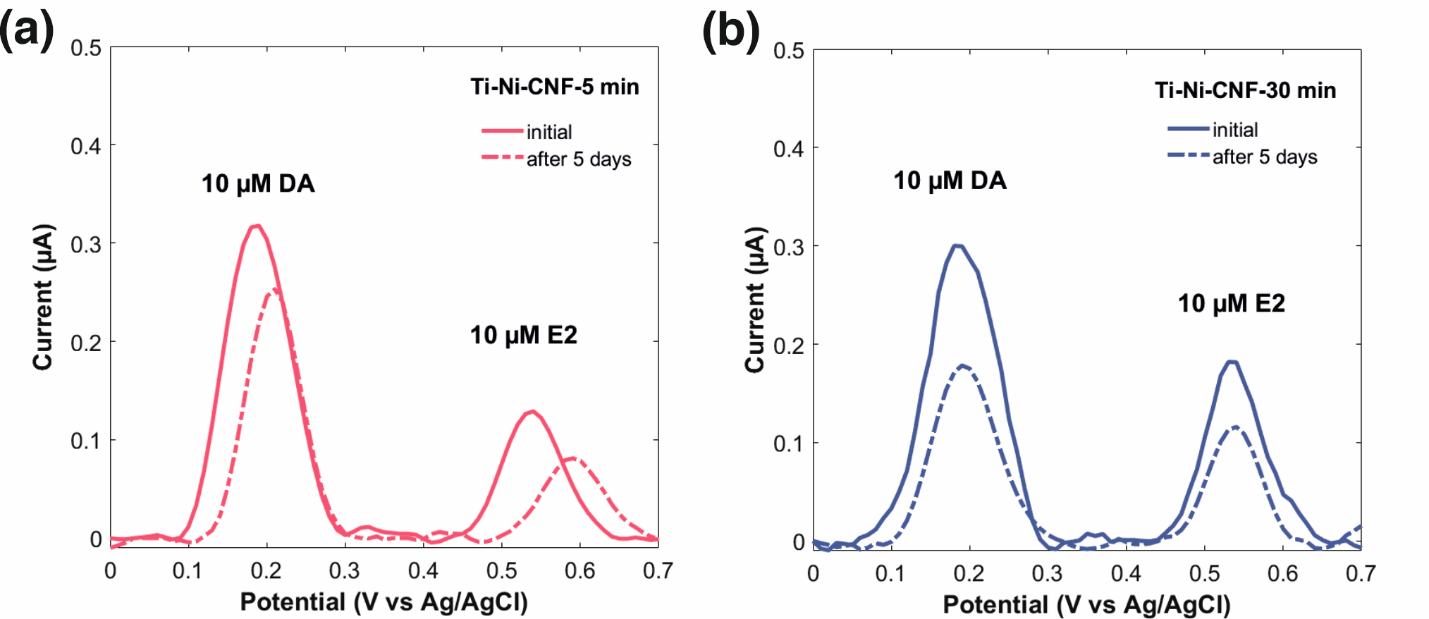


**Fig. S4** DPV responses of 10 µM DA and E2 in PBS pH 7.4 at 0.1 V pulse amplitude in representative **a** Ti-Ni-CNF-5 min and **b** Ti-Ni-CNF-30 min electrodes within a 5-day interval
